# Missed Opportunities for Universal HIV Screening in Primary Care Clinics

**DOI:** 10.4021/jocmr1014w

**Published:** 2012-07-20

**Authors:** Angela L. Hudson, MarySue V. Heilemann, Michael Rodriguez

**Affiliations:** a School of Nursing, University of California, Los Angeles, 700 Tiverton Avenue, Los Angeles, CA 90095, USA; b Department of Family Medicine, University of California, Los Angeles, 10880 Wilshire Blvd, Ste 1800, Los Angeles, CA 90095, USA

**Keywords:** Primary care providers, HIV testing, Ambulatory care

## Abstract

**Background:**

The Centers for Disease Control and Prevention (CDC) has reported that the number of new cases of HIV infection has been underreported annually by at least 40,000 cases. In 2006, the CDC recommended that voluntary HIV counseling and testing (VCT) was given to all patients aged 13 to 64 years in ambulatory care settings. The purpose of this qualitative study was to explore primary care providers’ (PCP) perspectives on and experiences of facilitators and barriers to offering VCT as part of annual screening.

**Methods:**

This was a descriptive, exploratory study where fifteen primary care providers were individually interviewed. Only community-based primary care providers were interviewed, and no obstetrician/gynecologists were enrolled, as VCT is standard of care in that specialty.

**Results:**

Barriers included doubts about the CDC recommendation, time constraints, fear, and assumptions about age and marital status. Facilitators included normalizing HIV testing and the availability of resources and training. PCPs’ role as an advocate and their professional style had the paradoxical potential of being both a barrier and a facilitator to VCT. Providers’ ability to connect patients to community resources was linked to their persistence and experience.

**Conclusions:**

Findings suggest more effort is needed by PCPs to facilitate HIV counseling and testing more frequently to their ambulatory care patients.

## Introduction

The number of persons acquiring HIV infection in the United States is estimated to increase by 40,000 to 56,000 cases annually (Centers for Disease Control and Prevention [1]). According to the CDC, approximately 1.1 million persons in the United States are infected with HIV; however, 25% of those who are infected do not know their HIV status and remain undiagnosed [1]. In 2006, the CDC recommended that primary care providers offer routine, voluntary HIV counseling and testing (VCT) as part of standard medical care to all patients aged 16 to 64 years, regardless of the patient’s risk behavior or pregnancy status [2]. However, the estimates of those undiagnosed with HIV remains high, which reflects underutilization of testing [1, 3]. Undiagnosed HIV in individuals can rapidly progress to a diagnosis of AIDS as a result of succumbing to opportunistic infections or receiving the diagnosis so late in the course of infection that they are unable to fully participate in beneficial early treatment and support modalities [4]. As a public health strategy to promote universal VCT, the CDC no longer requires signed consent with testing and has proposed an “opt out” model that assumes testing and counseling will be offered by the provider, although the patient can decline [4].

For pregnant women, routine universal VCT has life-saving benefits; VCT facilitates implementation of antiretroviral medication for pregnant women who test positive, thus decreasing mother-to-child HIV transmission [5]. Since testing has been a part of standard prenatal care since 2001, the incidence of perinatally-acquired HIV infection has declined significantly to less than 2 percent [4]. In fact, the AIDS Clinical Trials Group Protocol 076 in the United States demonstrated a reduction of mother-to-child vertical transmission of HIV from 25.5% to 8.3% using zidovudine alone [6]. Other studies in the United States have shown mother-to-child transmissions rates were reduced to as little as 0-1% among HIV-positive pregnant women receiving highly active antiretroviral therapy [7-9].

Despite evidence of the importance of screening, physicians’ beliefs and practices can create powerful obstacles to testing. In 2002, Troccoli and colleagues surveyed prenatal care providers about their practice of offering VCT and found that .the most significant barriers were related to managing the care of an HIV positive patient such as the provider’s feelings of discomfort with having to inform a patient she is HIV positive. Other researchers found barriers to ordering VCT to include inadequate reimbursement, discomfort with discussing risk behaviors with patients, and competing priorities in terms of what goals the provider had for a particular patient visit [10-12]. While these findings signal problems with VCT, no qualitative inquiry has been implemented to date to gain a more nuanced understanding of providers’ perceptions or concerns related to such barriers.

Despite the removal of the requirement for informed consent by the CDC in 2006, many individuals reported that they were not offered the test in a 2009 study [13]. Little is known about what kinds of barriers remain from the perspective of providers related to testing for the general population, targeting those between age 13 and 64.

Nonetheless, research done after the initiative has shown that when primary care providers (PCPs) take time to educate and counsel their patients about the importance of knowing their HIV status, patients are more receptive to HIV testing. Bassett and colleagues found that when on-site testing was offered to patients in an ambulatory clinic who had already received counseling about the importance of knowing their HIV status, an average of 39 new HIV cases were identified per week compared with an average of 8 new cases per week when patients were referred by their physician to a hospital lab [14]. Even adolescents will accept VCT when offered [15, 16]. Both provider-initiated and routine VCT leads to higher rates of testing acceptance and detection of HIV disease. However, high-risk patients, such as individuals with tuberculosis (TB), still go without guidance about HIV testing from their physicians, which is especially problematic for this high-risk population [17]. Rodger and colleagues found that nearly one-half of patients with TB (n = 1941) in their sample were neither counseled nor offered HIV testing. However, when these patients were later given information on the importance of HIV testing, 75% accepted the invitation to be tested [18].

Because PCPs are in an optimal position to offer VCT, insight can be gained by exploring their experiences with and perspectives on routine testing in the context of the CDC initiative. The first aim of our study was to explore PCPs’ perceptions of what serves as a barrier and what facilitates their ability to offer routine VCT to their patients. The second aim of this study was to explore PCPs’ reflections on their confidence and abilities to link newly diagnosed HIV positive patients to community support services.

## Methods

This is an exploratory, descriptive study guided by the techniques of Grounded Theory [19, 20]. After receiving university institutional review board approval, the principal investigator (PI) contacted 42 PCP practices that employ 238 PCP’s in the Los Angeles area by phone and mail between April 2009 and April 2010. A total of 15 PCPs agreed to participate in the study and all met eligibility criteria as licensed PCPs in the state of California (medical doctor, doctor of osteopathy, nurse practitioner, or physician’s assistant) who practiced within Los Angeles County, provided primary ambulatory care to adults or children, were able to speak English, and did not practice in a specialty area of medicine, such as cardiology, neurology, psychiatry, etc.

Each participant engaged in one individual interview led by the PI; the interview lasted between 30 - 60 minutes. Participants responded to questions based on a semi-structured interview guide. The interview was audio recorded and transcribed verbatim. Each participant received a $50 gift card at the conclusion of the interview.

Data were analyzed using Grounded Theory techniques [19]. Initially, each transcript was read comprehensively, then coded line-by-line, followed by focused coding to identify categories and subcategories. Memos were written to explore and describe emerging categories as well as relationships between categories.

## Results

### Study population

The 15 participants practiced in clinical areas of family practice, internal medicine, or pediatrics. The mean age of participants was 37.13 (SD = 8.59). More female PCPs participated than males (Table 1). All PCPs spoke English and some were bilingual in languages such as Spanish, Russian, or Chinese. PCPs had different levels of clinical experience and the populations served ranged from adults and teens, to children and families. Several PCPs also happened to provide prenatal care to pregnant women. Only three PCPs in our sample reported ever having diagnosed HIV in their current practice.

**Table 1:** Characteristics of Primary Care Providers

	N = 15
Race/Ethnicity	
African American	3
Caucasian	8
Asian/Pacific Islander	3
Hispanic	1
Clinic Type	
Public	4
Private	10
School-based	1
Age	
Sex	37.13 (SD = 8.59)
Male	6
Female	9
Clinical Specialty	
Family practice	9
Pediatrics	3
Internal medicine	3
Professional Affiliation	
MD	12
Nurse practitioner	1
Physician assistant	2
Average N Pts Seen Daily	24.7 (SD = 11.7)
Average N Pts Requesting HIV Test per month	4.33 (SD = 7.96)

### Using the study interview as an intervention: a rare opportunity to think out loud

The research interviews for this study were designed to collect descriptive data from PCPs, however, the interviews inadvertently served as a type of intervention. That is, the time that the providers spent in dialogue with the researcher seemed to be received by the PCPs as an opportunity to think out loud with an informed colleague, in private, about a topic they had rarely or never discussed to any significant extent, i.e., VCT and the CDC initiative.

About half of the providers in our sample had never heard of the initiative until approached by the PI. One said, “I actually found out about it from you, and I think it’s a good idea” and another PCP said, “I think you’re the first person I heard it from, but I think it’s good.”

The research interview was received as an opportunity to discuss various issues of HIV testing with a trusted, open-minded, interested and knowledgeable colleague. The interview and the PI’s attentive presence seemed to create a dialogical space in which providers were able to play devil’s advocate with themselves, to analyze their own thoughts verbally, to hear themselves articulate their own views in relation to the CDC initiative, to formulate and then ponder their own questions, and to describe their own practice related to VCT. The PCPs often gained insight as the interview progressed. Many providers grappled with system issues during the interview, voicing and analyzing the system of the clinic site where they worked. This included what was beneficial at their site and what could be changed. Many PCPs realized after the interview that they had become much more aware of the details and meaning of the initiative as a result of exploring their own views in the interview.

### Factors influencing use of VCT

The PCPs’ use of VCT for their patients was influenced by four types of barriers (doubts about the initiative, time constraints, fear, and assumptions about age and marital status) and two types of facilitators (normalizing VCT and resources such as training). Additional elements had the potential of being both a barrier and a facilitator to VCT (advocacy and professional style). Finally, the provider’s ability to connect patients to community resources was linked to their experience, their staff, and their ingenuity to persist in following up with patients.

### Barriers to offering VCT

#### Doubts about the CDC recommendation

Providers readily spoke about their protocols for testing and about dilemmas they faced. For some, this brought up inner conflict related to a cost-benefit analysis of testing. One provider said that he wanted to see “data” that showed outcomes that “proved” that testing was “necessary” before he would be comfortable ordering the test routinely. Another raised doubt that the CDC could enact the initiative as policy and went on to discuss how she was unsure of how important it was to follow a CDC recommendation in general. A pediatrician remarked that since he had not heard about the initiative from the American Pediatric Association, he did not trust it.

Some participants wrestled with the possibility that if testing was done routinely, patients might think that the test was being ordered due to a profit motive on behalf of the doctor or the clinic. Others questioned how a compulsory screening would influence the trust of the patient for the provider; “I don’t know about making it mandatory, though, because anytime you make someone do something, it’s going to set them up for not wanting to trust you.” One provider discussed her practice of ordering an HIV test for patients but was emphatic during the interview that she only ordered the test when it was warranted clinically, but not because of any other reason. She spoke at length about how she was not guided by a drive to simply please a patient by ordering whatever tests they wanted but rather by a desire to “help them and prevent anything that could be going on” in relation to sexually transmitted infections.

#### Time constraints

Providers expressed concern about the amount of time required to provide HIV counseling and education before ordering an HIV test. Likewise, completing the necessary paper work for obtaining consent was considered to be very time consuming. Some providers clearly were not aware that the CDC no longer required written consent and expressed concern about a reduction in productivity due to the need to spend “so much time” going over informed consent with the patient. A common rationale involved cost; “our reimbursement is too low to have too much time spent with patients.”

In contrast, some PCPs expressed relief that at their site, ordering an HIV test only required a simple check mark on the lab requisition form without counseling the patient. Others wished for such an easy routine for ordering HIV tests. One provider stated he would have no problem ordering an HIV test on every patient if this was the case, as long as he did not have to deal with the time involved with educating the patient as to why he was ordering the test.

#### Fear combined with avoidance, trust, or stigma

Providers expressed fear of some aspects of dealing with VCT which was related to avoidance, trust, or stigma. Avoidance of testing was demonstrated in various ways by different providers. One provider admitted that, in fact, he really did not know what to do when a patient tested positive for HIV, so he avoided ordering the test. Others expressed uncertainty about how to deal with the emotional aspects of informing a patient that their test was positive which they said, in the long run, could make them avoid testing. Many providers felt that avoidance of testing would be reduced if there has been an HIV counselor on site to do the education and counseling, because this would reduce the “burden” of the time-consuming interaction with the patient.

Almost half of our sample was concerned about the ability of the patient to trust them. Some felt that mandatory testing was not conducive to a trusting relationship between provider and patient because patients might feel coerced into getting tested. In addition, some providers voiced awareness that patients feel stigma about getting tested. Several expressed empathy for patients who felt “condemnation” or “shame and guilt” about the reason they needed to be tested. Providers wanted to help reduce these negative feelings for patients so that they would get tested without emotional turmoil. PCPS pointed out that stigma was reduced by celebrities who used media to advocate for getting tested because they acted as role models who motivated patients to get HIV testing.

#### Assumptions about age and marital status

Providers’ perceptions about age played a part in the decision to offer or order an HIV test to elders and teens. Some PCPs automatically assumed that older patients (aged 60 or 70 years) were not at risk simply because of their age. This bias was not identified as a problem by the providers, but simply a way of making the decision to test or not to test.

Likewise, assumptions about what it meant to be a “younger” patient were plentiful among our sample, although perceptions about attributes associated with being a “younger” patient were different for different providers. Most providers assumed their teen patients were sexually active and tended to offer and order HIV tests for all teens regardless of their sexual history. The assumption that adolescents are sexually promiscuous was made by several PCPs which led some providers to wonder if sexually active teens should be tested every six months. In contrast, other providers wanted additional information about their teen patients in order to make their decision to test. Some needed to know a teen had had sex more than once while others needed to know that the teen was in an on-going sexual relationship. A pediatrician spoke about the impracticality of offering universal HIV testing to non-sexually active teens because many are just simply “not engaging” in sexual relationships currently.

In addition, issues related to ethics were raised such as the morality of testing a person as young as 13 years of age even if they were sexually active. The issue of parental knowledge of the sexual activity of their teenage son or daughter was a complex issue that served as an additional stumbling block to testing for many providers.

Having married patients seemed to deter some PCPs from offering those particular patients VCT. PCPs reported that their married patients engaged in a reasoning process that eradicated their sense of risk; since their status changed to that of a married person, they seemed to automatically assume that they were not at risk. Acknowledging that the view that all married people are monogamous involved a naive assumption, one PCP stated that even if he does ask about their sexual activity, patients may or may not tell the truth. Indeed, no provider reported asking any married patient if they had had other sexual partners. However, one PCP acknowledged he will offer the test if a married patient reports having had other sexual partners. “In general, I’ll offer it if they want it. If they feel they need it, I’ll kind of go through sexual education, if they’re not using condoms. If you’re married for so many years it probably doesn’t really matter, you know? It’s up to them, like if they’ve had.....because you know, and they don’t always tell you if they’ve had other partners.”

### Facilitators for offering VCT

#### Normalizing VCT

Providers discussed the high prevalence of chronic illnesses in their respective practice areas, especially among adult patients, and stated that screening for HIV should be no different than screening for glucose level, cholesterol level, or blood pressure. They reported a need to give “a lot of time-consuming support” to patients with conditions such as diabetes mellitus, hypertension, and cardiovascular disease far more often than for patients with HIV infection. These providers considered HIV infection to be a chronic disease and that patients should be aware that insurance companies will cover the cost of HIV screening, just as they cover the cost of mammography screening, prostate screening (PSA(prostate specific antigen)) or colonoscopy screening. Regarding adolescents, a nurse practitioner who works at a school-based clinic reported that students, aged 13 years and older (and their parents) should expect screening for HIV just as they expect their children will be screened for tuberculosis and hepatitis for school registration and entrance purposes.

#### Resources and training

PCPs valued staying current with medical protocols and opportunities for training on what to do when a patient tests positive for HIV. While many admitted they had very little experience diagnosing patients with HIV, they did have some experience working with patients who had been diagnosed elsewhere. Providers clarified that they felt confident about their clinical skills but their confidence regarding HIV waned in terms of what to do after the test was ordered. They remembered being trained in medical school and residency on how to counsel patients and stated that they were aware of California law related to confidentiality for minors. But, providers felt challenged by what seemed to be “constantly changing” protocols for HIV testing. They hoped for more continuing medical education (CME) offerings to bring them up to date on VCT.

Providers expressed positive regard for resource materials to facilitate HIV testing. They wanted to have patient education materials and resources. Among the items identified as needed by various PCPs was a concise list of talking points to educate patients in “five minutes or less,” posters for exam rooms specifically tailored to facilitate HIV testing for 13-to-17-year-old patients, a resource list of HIV clinics and facilities in the area, and an HIV counselor on site for educational support.

Providers agreed that the media might be an effective resource for facilitating HIV screening. They cited the direct marketing of medications as enhancing their patients’ assertiveness regarding medication requests, and likewise, suggested that media campaign messages about the importance of knowing one’s HIV status could be influential. One PCP endorsed ads that linked sexual activity and HIV infection. She said, “I think this would make patients more aware of STDs, because they need to be reminded that risky behavior has results.” Another provider discussed his own discomfort with raising the issue of HIV testing with his patients and felt that festivals and health fairs could encourage patients to take responsibility for their health by requesting HIV testing and information from their physician.

### Both a facilitator and barrier to offering VCT

#### Professional style and communication

Providers described various aspects of their professional style for interacting with their patients and most reported a high regard for making the patient feel comfortable with HIV testing. They felt that the decision-making process is an important time to be the patient’s advocate and they reported a high value for patient autonomy when they offered an HIV test, especially if they did not offer testing on a routine basis. One provider emphasized his dedication to respect for patients when he demonstrated how he usually begins an HIV screening discussion. He explained how he typically begins a discussion, ‘Let’s go through some of the routine healthcare issues that I’d like to discuss in depth with you.’ That way you have the patient motivated. When I offer a patient an HIV test, I say, ‘Can I add this to your list of tests?’ And just see how the patient feels, if they say ‘yes’ or ‘no’. If they say ‘no’, then I might consider talking about it more in depth at another visit.

In addition to a show of respect, another matter of professional style was engaging in honest communication during the patient visit. A pediatrician reported that patient honesty was the product of “a good physician-patient relationship” built on the physician’s genuine sense of caring for the patient. With a firm anchor in honest, straightforward communication, obtaining a comprehensive sexual history was an important standard of practice adhered to by many PCPs. The sexual history helped providers identify high-risk behaviors. One PCP reported initially engaging the patient in a non-threatening history-taking activity, such as asking adult patients about dating or inquiring about school activities with teens, then inquiring about boyfriends or girlfriends, followed by questions about sexual activity. This provider stated that such a beginning created a nice segue to the discussion of sexual behavior followed by the topic of HIV screening.

Being empathetic to patients’ feelings about getting tested for HIV also was an important aspect of how some providers governed their own professional style when interacting with patients. In terms of decision-making, the emphasis was on choice rather than mandated screening, which encouraged educational interactions to help the patients become involved with their own health choices.

Providers who had past experience with giving a patient a HIV positive diagnosis found that the patient needed time to digest the news. One provider reported that she would typically stay with the patient, i.e., spend time with them to counsel them and discuss the diagnosis. To demystify the diagnosis, she explained that HIV infection should no longer be perceived as “an immediate death sentence.” She gave other information about HIV and she allowed extra time for the patient to ask questions.

Although such aspects of a PCP’s professional style could facilitate testing, it could also serve as a barrier to VCT. For example, one provider stated that he encouraged his patients to go to an anonymous test site rather than get tested during an office visit with him. That is, instead of ordering the test himself, this PCP recommended that his patients seek testing at a free-clinic where their anonymity would be protected. Without indicating any awareness of the missed opportunity to test at his own clinic, and without regard for the potential that his patients may not actually ever follow through with testing at an anonymous site, this PCP reasoned that patients might be able to accept their diagnosis and treatment more easily if a stranger gave it to them.

Some providers followed self-imposed rules that limited VCT. For example, some would order a test only if the patient reported a history of intravenous drug use or if the patient reported several past/current sexual partners. Another provider who worked with teens at a school-based clinic admitted not ordering HIV testing because “teens do not like needle sticks.”

#### Advocacy and the limits of testing

Issues of advocacy could either facilitate or hinder a provider from ordering an HIV test. For example, PCPs who viewed themselves as a patient advocate were more likely to report talking with and testing teens if they were sexually active; however, they were less likely to order the test if they could not assure confidentiality for that teen. Unless they had full confidence that the teen’s confidentiality would be protected completely (especially from their parents), some providers stated they simply would not test. One provider considered it a breach of confidentiality that California law requires providers to inform parents of teens, younger than aged 14 years, if they are tested for HIV.

### Linking patients to HIV/AIDS support services

Providers were at different levels of readiness to link patients to support services. Some reported having no experience in this area at all and very little knowledge about where to refer patients after a positive diagnosis. Others were aware of support programs that existed in their communities and were proud of the fact that their clinic had a comprehensive list of HIV care clinics available, but they also reported that they had no experience using these services or referring any patients to them. Only two providers said that they were experienced with making referrals. Rather, it was their staff who received training and acted as a team, using strong connections to community agencies, that offered social support services. However, one PCP designed a unique follow-up visit protocol based on evidence from the literature that patients from low-income populations often fail to follow-up after HIV diagnosis. This PCP set up a special follow up appointment with the patient after they completed the first visit to the infectious disease clinic post HIV diagnosis to be sure that they were connected to care including ancillary services and following through with needed treatment.

## Discussion

Very few PCPs in our sample (3 of 15) reported that they had diagnosed HIV in their current practice and only two reported that they were currently offering VCT as standard of care. Thus, our sample provided ample input on what barriers contributed to missed opportunities for VCT in primary care sites but less input on what enhanced PCPs to offer VCT. Our results showed that the actual process of offering and ordering an HIV test for patients involved a facilitator-barrier dynamic fueled by a variety of factors.

A societal barrier, that served to discourage testing, included doubts about the CDC recommendations for testing, especially HIV testing of adolescents. Several of our participants did not know that written, informed consent was no longer required. This raises the issue of which professional organization different groups of PCPs look to for their clinical guidelines. For example, if the CDC is not the most trusted authority, perhaps particular professional organizations such as the American Academy of Pediatrics or the American College of Physicians: Internal Medicine could formally endorse the CDC guidelines and make their endorsement widely known to their members. Such a demonstration of professional solidarity on the issue may enhance providers’ comfort with the initiative and might it may be more effective in relation to actually changing the practice of PCPs to implement CDC testing recommendations.

Similar to the findings of the survey research done by Troccoli and colleagues’ with prenatal care providers, our data show that the major barriers to offering VCT were problems with obtaining informed consent, difficulty deciding whom to test, and concern about offending the patient by offering the test [20]. While none of the PCPs in our sample seemed to take the issue of HIV testing lightly, several factors made us aware that the PCPs were in need of reliable information about HIV and CDC guidelines. Not only were they under-informed about the initiative and reluctant to trust the CDC, but they were eager to discuss the initiative with the study PI and voiced a desire for more training on the issue.

Facilitating factors encouraged testing such as the ability to consider HIV another chronic disease for which any PCP should test. This act of normalizing HIV could, theoretically, lead a PCP to assume HIV deserves a standard protocol to assure routine testing for all patients. Indeed, offering HIV testing as part of a standard screening protocol holds promise to reduce the stigma of testing. In support of this, Brooks and colleagues found that routine offering of HIV testing was capable of becoming an expected and accepted practice by patients and clinic staff [21]. They pointed out that just as lab results might induce persons to eat more healthfully, lose weight, and exercise regularly, individuals might engage in safer sex practices if they know their HIV status based on annual screening.

PCPs were forthcoming in their desires for VCT resources, which they indicated were very limited or non-existent. Through the interview process, many PCPs became aware that they were missing opportunities to offer the HIV test. Training was desired by the providers and could address the purpose of having standard protocols for VCT, the importance of following-up with patients, and ways to avoid missing opportunities for testing. PCPs’ concerns that patients would not trust the provider if the screening was mandatory underscores the need for PCPs to work through emotions that interfere with their ability to assess HIV risk [10]. Bokhour and colleagues found that providers were more amenable to offering VCT when they were informed that patients can opt-out of HIV screening, when they realized that patients do not need to sign a separate consent form, and when a simple statement was made to the patient that an HIV test will be included in their screening panel [22]. This provides evidence that resources specifically tailored to facilitate providers’ ability and willingness to offer VCT are effective.

Without hesitation, the PCPs of our sample confided that they wanted patients to be ready-to-learn when they enter the exam room. In their views, waiting room materials were needed such as prompts, posters, or pamphlets that would catch the patient’s attention and prepare the patient for discussing HIV during the health visit. Thus, efforts focused on the development and dissemination of materials that are specifically designed to both engage and inform patients would be an important step to help the patient to be ready to discuss VCT. This holds the potential for increasing PCPs’ confidence to bring up the topic during the visit.

In some cases, facilitators and barriers combined in a unique way to influence the PCPs’ use of VCT. These factors included the individual communication or professional styles of each provider as well as their interpretation of what it meant to advocate for a patient’s rights (specifically, confidentiality). According to Hanssens, primary care providers need informational support through adequate training in how to offer VCT [23]. She has suggested that providers engage with local AIDS service organizations to gain the knowledge needed to be able to embrace the CDC guidelines.

With our sample, there was an uneven level of comfort and savvy regarding bringing up the topic of sex. Some were comfortable but others struggled. Patients younger than 13, those who were married, those without symptoms, and those without a injection drug use history were problematic for different providers in terms of ordering VCT. Reluctance to offer VCT may be strengthened by particular assumptions made or stereotypes held by a particular provider. PCPs need experience in order to gain confidence and to enhance their skills for interviewing patients about sexual behavior without feeling they are insulting or shaming the patient when raising the issue of VCT.

Again, the majority of PCPs reported discomfort or inexperience linking newly-positive HIV infected patients to social services. Although one PCP specifically designed a protocol to ensure that her newly diagnosed HIV positive patients were given an appointment with the infectious disease clinic and subsequent follow-up appointment with her to see how things went, most PCPs had no experience with patients who were newly-diagnosed in their respective practices. This highlights the possibility of a variety of missed opportunities for VCT. Clearly education, training, and discussion are needed for PCPs to overcome discomfort with the topic of HIV and sexual behavior, to gain experience, and to reduce the number of missed opportunities for VCT.

### Study limitations

Qualitative research is useful for gaining a nuanced understanding of the particular experiences of a sample of participants related to a phenomenon. As such, findings from a study like ours cannot be generalized to all primary care providers or to all settings. We purposely did not include obstetricians and gynecologists in the study because VCT is standard of care in that specialty area. However, it is possible that their perspectives might have added further insight into VCT. A longitudinal design with multiple interviews over time would have enhanced our potential insight into this phenomenon.

### Conclusion

According to CDC recommendations, VCT is to be offered to all patients, ages 13 to 64, in primary care settings, irrespective of a patient’s presenting complaint [4]. In order for VCT to be a successful public health initiative, PCPs need to know that time-consuming pre-counseling sessions are not required and verbal consent is sufficient [24].

Post-test counseling is to be conducted to ensure the patient understands the test result and receives prevention instruction, while newly diagnosed HIV-positive patients must be linked to appropriate treatment, care, and support services [25].

The strongest predictor of HIV testing is when the PCP encourages it [26]. When the majority of primary care providers begin to integrate HIV testing as part of standard practice, fewer missed opportunities for HIV case finding are likely. Even when prenatal testing began as standard of care, providers were basing testing decisions on their assessment of a pregnant woman’s risk status [20]. The gap between what is the recommended protocol and what is the reality cannot be bridged unless PCPs are provided with education, training, and opportunities to discuss the issues related to VCT with trusted, knowledgeable colleagues. Not only the patient, but whole communities, ultimately benefit from early diagnosis and treatment.
